# A stochastic B cell affinity maturation model to characterize mechanisms of protection for tetravalent dengue vaccine constructs

**DOI:** 10.3389/fmolb.2023.1100434

**Published:** 2023-07-14

**Authors:** Venkat R. Pannala, Hung D. Nguyen, Anders Wallqvist

**Affiliations:** ^1^ Department of Defense Biotechnology High Performance Computing Software Applications Institute, Telemedicine and Advanced Technology Research Center, U.S. Army Medical Research and Development Command, Frederick, MD, United States; ^2^ The Henry M. Jackson Foundation for the Advancement of Military Medicine, Inc., Bethesda, MD, United States

**Keywords:** dengue infection, tetravalent vaccines, immune response, stochastic simulations, humoral immunity

## Abstract

Dengue annually infects millions of people from a regionally and seasonally varying dengue virus population circulating as four distinct serotypes. Effective protection against dengue infection and disease requires tetravalent vaccine formulations to stimulate a balanced protective immune response to all four serotypes. However, this has been a challenge to achieve, and several clinical trials with different leading vaccine candidates have demonstrated unbalanced replication and interference of interindividual serotype components, leading to low efficacy and enhanced disease severity for dengue-naïve populations. Production of serotype-specific neutralizing antibodies is largely viewed as a correlate of protection against severe dengue disease. However, the underlying mechanisms that lead to these protective immune responses are not clearly elucidated. In this work, using a stochastic model of B cell affinity maturation, we tested different live-attenuated vaccine constructs with varied viral replication rates and contrasted the initiation and progress of adaptive immune responses during tetravalent vaccination and after dengue virus challenge. Comparison of our model simulations across different disease-severity levels suggested that individual production of high levels of serotype-specific antibodies together with a lower cross-reactive antibody are better correlates for protection. Furthermore, evolution of these serotype-specific antibodies was dependent on the percent of viral attenuation in the vaccine, and production of initial B cell and T cell populations pre- and post-secondary dengue infection was crucial in providing protective immunity for dengue-naïve populations. Furthermore, contrasting disease severity with respect to different dengue serotypes, our model simulations showed that tetravalent vaccines fare better against DENV-4 serotype when compared to other serotypes.

## Introduction

Dengue is an arboviral disease caused by four serotypes of dengue virus (DENV 1–4). The virus, which is transmitted by mosquitos, is estimated to infect several hundred million people each year living in tropical and sub-tropical regions around the world (Franco et al., 2010; Bhatt et al., 2013; Schaffner and Mathis, 2014). A primary infection with a DENV serotype results in long-term immunity against the same serotype but may only provide partial protection against other serotypes (Montoya et al., 2013; Guzman and Harris, 2015). Primary DENV infections are typically asymptomatic, with only a minority of patients exhibiting mild, uncomplicated dengue fever. Following primary infection, production of serotype-specific antibodies, which circulate for decades if not longer, are known to provide protection against the homologous serotype in the event of a secondary subsequent infection (Halstead, 1974; Wahala and Silva, 2011). However, infection with a second DENV infection of a different serotype (heterotypic) leads to a greater risk of developing severe dengue hemorrhagic fever and shock syndrome. One hypothesis for such an enhanced severity with the heterotypic infections is that some of the cross-reactive antibodies developed during the primary infection promote the entry of the heterotypic virus into Fc receptor-bearing target cells as the initiating event leading to severe disease (Halstead, 2003; Rothman, 2011; Katzelnick et al., 2017). Therefore, the development of vaccines for dengue infections is not straightforward and requires polyvalent vaccine formulations to induce a simultaneous and balanced protective immunity against all four serotypes.

There are several candidate tetravalent dengue vaccines under clinical evaluation, including two live-attenuated DENV vaccines currently in phase III trials and one live-attenuated vaccine that has been licensed for use in children (Dengvaxia) with pre-existing immunity to DENV (Sabchareon et al., 2012; Capeding et al., 2014; Sridhar et al., 2018). However, Dengvaxia was poorly efficacious in children with no pre-existing immunity and increased the risk of severe disease upon exposure to wild-type DENV infections (Henein et al., 2017). Despite its tetravalent formulation, Dengvaxia elicited unbalanced replication of vaccine components, with DENV-4 being immunodominant compared to the other three serotypes in several studies (Guy et al., 2009; Morrison et al., 2010; Dayan et al., 2014). Similarly, Dengvaxia did not afford protection against symptomatic DENV-2 infection in subjects who were dengue-naïve before vaccination (Capeding et al., 2014; Villar et al., 2015). These observations indicate that for dengue-naïve populations, the production of vaccine components is not uniform across all four serotypes.

Although the current vaccine design constructs are based on tetravalent formulations to induce balanced protective immunity to all four serotypes, in practice, it has been challenging to achieve balanced replication and protective immunity with tetravalent DENV vaccines. Several factors, such as the role of virus replication rate in the live-attenuated vaccines, properties of neutralizing antibodies, and efficacy durability in different recipient groups (e.g., seronegative *versus* seropositive, different age groups), are not clearly elucidated (Thomas and Yoon, 2019). To this end, simulations of the immune system using sophisticated models of the affinity maturation process are useful not only for predicting disease severity, but also for allowing us to examine the origin and progress of dengue infections and for providing an opportunity to test tetravalent vaccine formulations with equal probability of their component production. In our previous work, we successfully applied these models to delineate the mechanisms initiating and sustaining adaptive immune responses during primary infections (Nguyen et al., 2021). We used the affinity maturation model to characterize the antibody responses that are responsible for protection against severe dengue disease and investigated the mechanisms underlying the shift from a protective primary response to a non-protective secondary antibody response.

In this study, we extended our B cell affinity maturation framework to study the effect of vaccines on DENV infections in dengue-naïve populations. Incorporation of viral growth kinetics in our previous effort (Nguyen et al., 2021) allowed us to use these models to simulate different live-attenuated vaccine formulations with varied viral replication rates in the vaccine. Using the disease manifestations defined in our previous study (Nguyen et al., 2021), ranging from non-symptomatic to symptomatic cases, we investigated the optimal viral replication rate for live-attenuated vaccine designs, elucidated the mechanisms of protective immune responses in non-symptomatic dengue-naïve populations, and addressed the role of different dose regimens in providing better protection for dengue-naïve populations compared to a single-dose regimen.

## Materials and methods

### Mathematical model of B cell affinity maturation

The B cell affinity maturation model contains three major components of the coupled viral, B cell, and T cell dynamics that account for affinity maturation process. We modeled B cell affinity maturation as a set of rate reactions, like chemical reactions, that describe the underlying immunological processes, such as virus binding, B cell activation by T cells, and B cell replication, etc. For example, the model captured the viral growth kinetics using first-order reactions for virus replication and intrinsic decay due to non-specific clearance processes in the body and second-order reactions for B cell- and T cell-mediated virus clearance. Similarly, we modeled the formation and decay of naive B cells, CD4^+^ T cells, and cytotoxic CD8^+^ T cells as zero-order and first-order reactions, respectively. We modeled virus-mediated stimulation of naive, stimulated, and memory B cells using second-order reactions (Chaudhury et al., 2014; Chaudhury et al., 2017; Nguyen et al., 2021). We provide a complete description of the model rate equations and the associated rate parameters in the Supplementary Material S1.

We used immunological shape space models to describe the antigenic relationship between each virus serotype, at the epitope level, with respect to B cell specificity and cross-reactivity (Smith et al., 1997; Woo and Reifman, 2012). In this model, each virus belongs to one of the four serotypes (DENV 1–4) and has its own specific genotype. Each virus serotype contains the epitope genotypes for four epitopes: PrM from the pre-membrane protein, fusion loop (FL), domain III (DIII), and hinge from the envelope protein (Supplementary Table S1). Each epitope (and paratope) is represented as a 20-character string made up of four unique characters. The sequence difference between all four serotypes of each epitope shows that the antigenic distance is 0 for the PrM epitope, 1 for FL, 4 for DIII, and 5 for hinge (Chaudhury et al., 2017). All sequences are listed in Supplementary Table S1, and their parameters of immunogenicity and clearance are listed in Supplementary Table S2.

Similarly, all B cells and antibodies in the system are denoted by a paratope genotype with a 20-character string made up of four unique characters. There are seven types of B cells in the system with paratype genotypes represented as naïve, germinal center, stimulated, activated, short-lived plasma, long-lived plasma, and memory, along with antibodies so they can be identified as either DENV specific or cross-reactive. Using the system of rate equations described above, we carried out stochastic simulations of affinity maturation by applying the Gillespie algorithm (Gillespie, 2007) as adapted by Woo and Reifman (Woo and Reifman, 2012) for modeling the immune systems and executed in Python. We provide the source code in the Github repository (https://github.com/BHSAI/B-cell-affinity-maturation-model) and all the relevant rate parameter values in the Supplementary Material S1 for reproducibility.

### Limitations of the affinity maturation model

To avoid complexity, in the current version of the model we did not include a detailed paratope genotype-based classification for T cells, instead they are described as an overall abundance of polyclonal CD4^+^ and CD8^+^ T cells. For further simplification, other subtypes of B and T cells, such as TFH or NKT cells, and other immune cells, such as dendritic cells and macrophages which present the encapsulated virus to the B and T cells, are not exclusively considered in this model formulation. This is a valid assumption as our model is focused on the B cell affinity maturation process, requiring only virus particles as input. Furthermore, our model includes the antibody response to dengue virus infection at the virus epitope level only for viral structural proteins PrM and envelope (E) proteins. Although other structural and non-structural proteins play a role in antibody-mediated virus neutralization, it is well known that the PrM and E proteins are the primary targets of DENV neutralizing antibodies and are the focus of current DENV vaccine development efforts (Thullier et al., 2001; Roehrig, 2003; Gromowski et al., 2008; Pierson et al., 2008; Gromowski et al., 2010; Smith et al., 2016). In addition, we assumed that all the antibodies that bind to virus are equally neutralizing depending on their binding affinity and we did not impose any specific constraints that indicate cross-reactive antibodies possess weak neutralizing capabilities.

### Model formulation and simulation conditions

For all simulations, we initially ran the model for 100 days to establish an initial population of naive B cells and T cells and allowed the stochastic model to reach an equilibrium. We considered the equilibrated state as day 0 for vaccination, and the conditions were set up to reflect the dengue natural infections observed in the clinic for the primary infections (Salje et al., 2018). To investigate production of a balanced immune response against the four serotypes, we assumed all individual natural serotypes of the DENV replicate at the same rate in the tetravalent vaccine constructs. To obtain a tetravalent vaccine formulation with a specific live attenuation rate, we altered the virus replication rate, the stimulation rates of T cell-mediated activation of stimulated B cells, and the stimulation rates of CD4^+^ T cell-mediated activation of CD8^+^ T cells along with antibody and CD8^+^ T cell-mediated virus clearance rates (Supplementary Table S3). For each simulation, we administered an initial virus dose of 10^5^ copies/mL of tetravalent vaccine at day 0 for single-dose administrations and subjected the population to a similar dose of natural DENV-2 exactly 1 year later. Similarly, for multiple doses, we administered a concentration of 10^5^ copies/mL of tetravalent vaccine at days 0, 90, and 180 and subjected the population to a DENV-2 challenge exactly 1 year after the last dose of vaccine.

We carried out 10,000 simulations for each condition, and each simulation produced a unique trajectory representing the outcome of a single individual. At the beginning of each simulation, all variables (concentration of B cells and T cells) were set to zero except for the concentrations of naive B cells, helper CD4^+^ T cells, and cytotoxic CD8^+^ T cells. We randomly chose these concentrations from respective non-normal distributions whose median, minimum, and maximum values were from a healthy population of children who were 6–12 years old (Tosato et al., 2015). Depending on the single or multiple vaccination protocol, the stochastic model for each condition was simulated for approximately 800–1,000 days, and each simulation lasted ∼1 h on a desktop computer. We report the average values with error bars denoting one standard deviation at each time point, as well as the overall distribution of outcomes.

## Results

### Model simulation of tetravalent live-attenuated vaccine constructs

**FIGURE 1:**
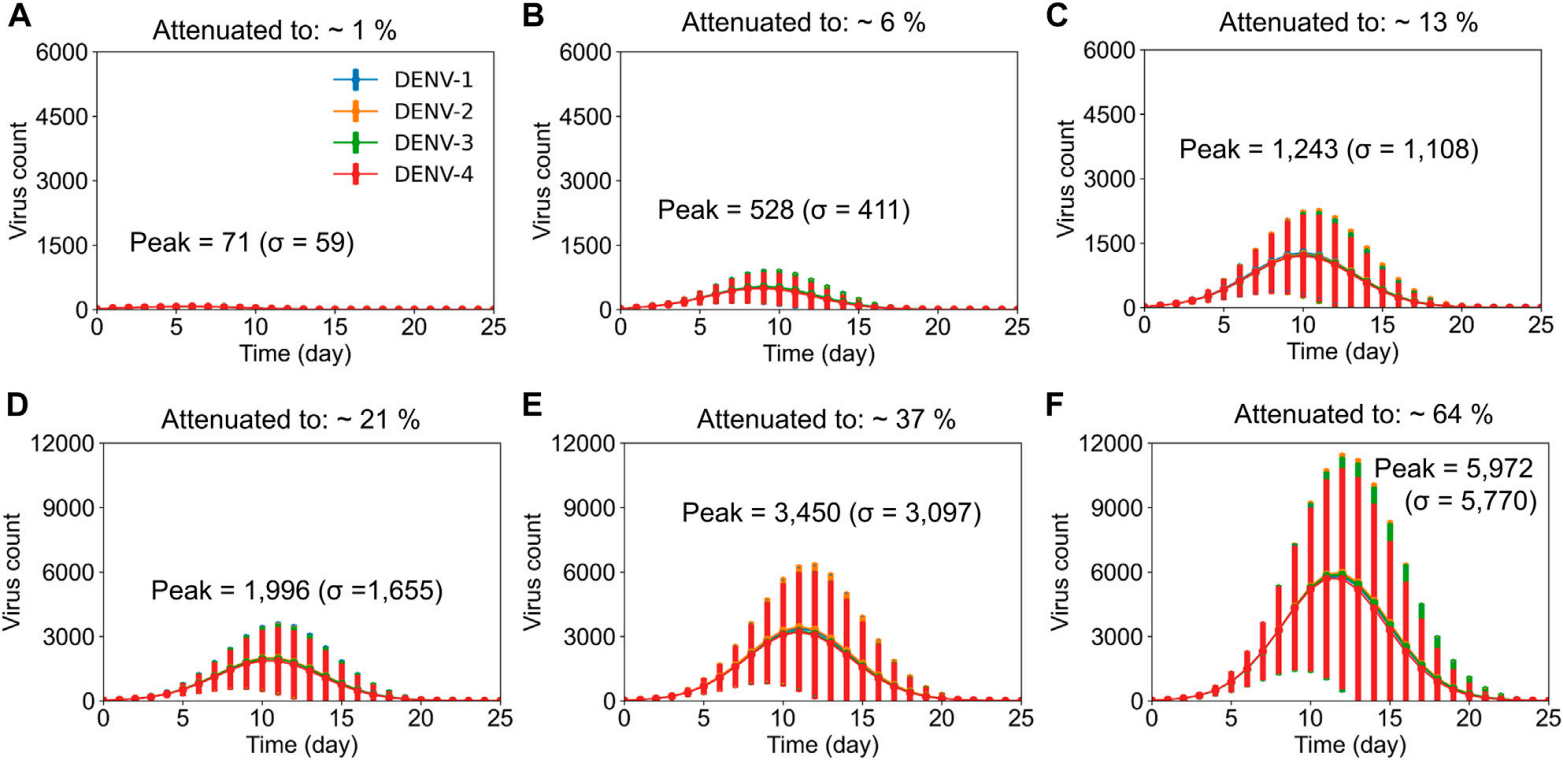
Stochastic model simulations of live-attenuated virus with different percentages of virus replication rate in the vaccine. Average concentration (units × 10^3^/mL) of virus particles is shown as a function of time post vaccination for all four DENV serotypes at different virus replication rates, with error bars representing population standard deviations (σ). It took an average of 10,000 simulations to achieve **(A)** ∼1%, **(B)** ∼6%, **(C)** ∼13%, **(D)** ∼21%, **(E)** 37%, and **(F)** 64% virus replication in the vaccine compared to virus replication with natural infections.

As exemplified in our previous work (Nguyen et al., 2021), our stochastic model captured the viral growth kinetics observed in laboratory investigations under primary natural infections (Tricou et al., 2011). Therefore, we used the peak viremia observed in those studies as the benchmark and altered the viral replication rates in the model such that we could obtain a reduced peak viremia in the system to represent the different live-attenuated virus activities. We assumed an equal replication rate for each virus serotype in the tetravalent vaccine and tested virus growth kinetics for different replication rates. Figure 1 shows the stochastic model simulations of the viral growth (average concentration of virus) with time when the virus replication rates and their clearance were altered compared to rates from natural primary infections. Compared with the viral growth in natural infections, where peak viremia was observed approximately on day 10 (Nguyen et al., 2021), virus attenuation led to altered growth kinetics with peak viremia shifted around day 10. When viral attenuation was stronger, we observed peak viremia earlier than day 10 (Figure 1, top panels) and the peak viremia achieved was less than 13% of that with natural infections. However, as the viral replication rate increased above 13% (Figure 1, bottom panels), the peak viremia shifted beyond day 10. We used these simulations as *in silico* live-attenuated vaccine constructs and evaluated the production of protective immune responses in dengue-naive populations against the DENV-2 challenge model.

### Evolution of protective immune responses to tetravalent live-attenuated vaccine constructs

**FIGURE 2:**
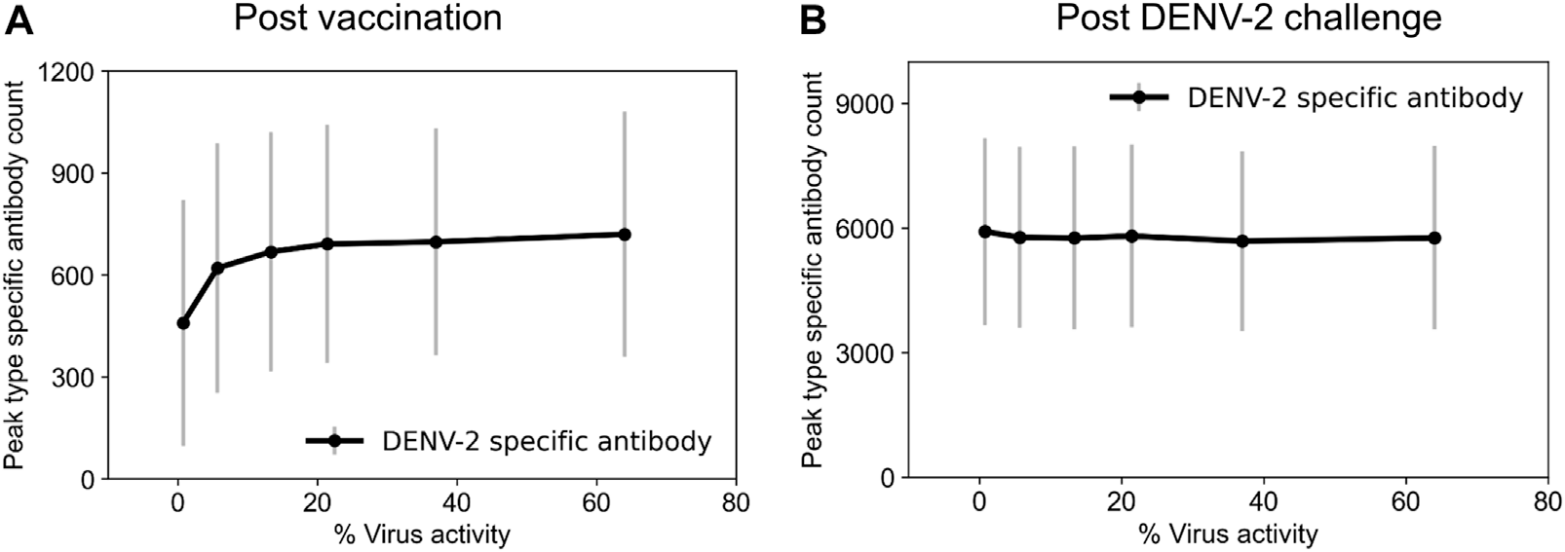
Stochastic model simulations of peak serotype-specific antibody production at different percentages of virus replication rates in the live-attenuated vaccine construct compared to natural infections. Peak DENV-2-specific antibody counts (units × 10^3^/mL) are shown **(A)** post vaccination and **(B)** post DENV-2 challenge. Here, we define virus activity as the ratio of peak viral count at a given virus replication rate to the viral count under natural DENV infections.

In the case of a primary infection (an individual infected with DENV for the first time), a person develops a protective serotype-specific antibody response that is correlated with resistance to reinfection by the same serotype. Therefore, for the dengue naïve population in our study (children between ages 6–12), we tested the production of serotype-specific antibodies for the different tetravalent vaccine constructs immediately upon vaccination following a single dose of vaccine administration (day zero). We observed a simultaneous production of serotype-specific antibodies for the four serotypes after vaccination that coincided with the virus growth, however, the time to peak antibody production was dependent on the level of virus replication in the vaccine (Supplementary Figure S1). For example, we observed peak antibody production at day 16 for 1% virus replication in the vaccine and at day 25 for 64% replication, and the antibody production reached basal levels approximately 100 days post vaccination in both cases. In addition, despite the same replication rates for each serotype in the vaccine construct, we observed that peak type-specific antibody production for DENV-4 is slightly higher compared to other serotypes. Furthermore, our model simulations also showed that the peak serotype-specific antibody production increased with increased virus replication in the vaccine and reached saturation around 13% virus replication (Figure 2A). These simulations showed the minimal virus replication that is required in the live-attenuated vaccines to produce protective immune responses in the form of serotype-specific antibodies.

**FIGURE 3:**
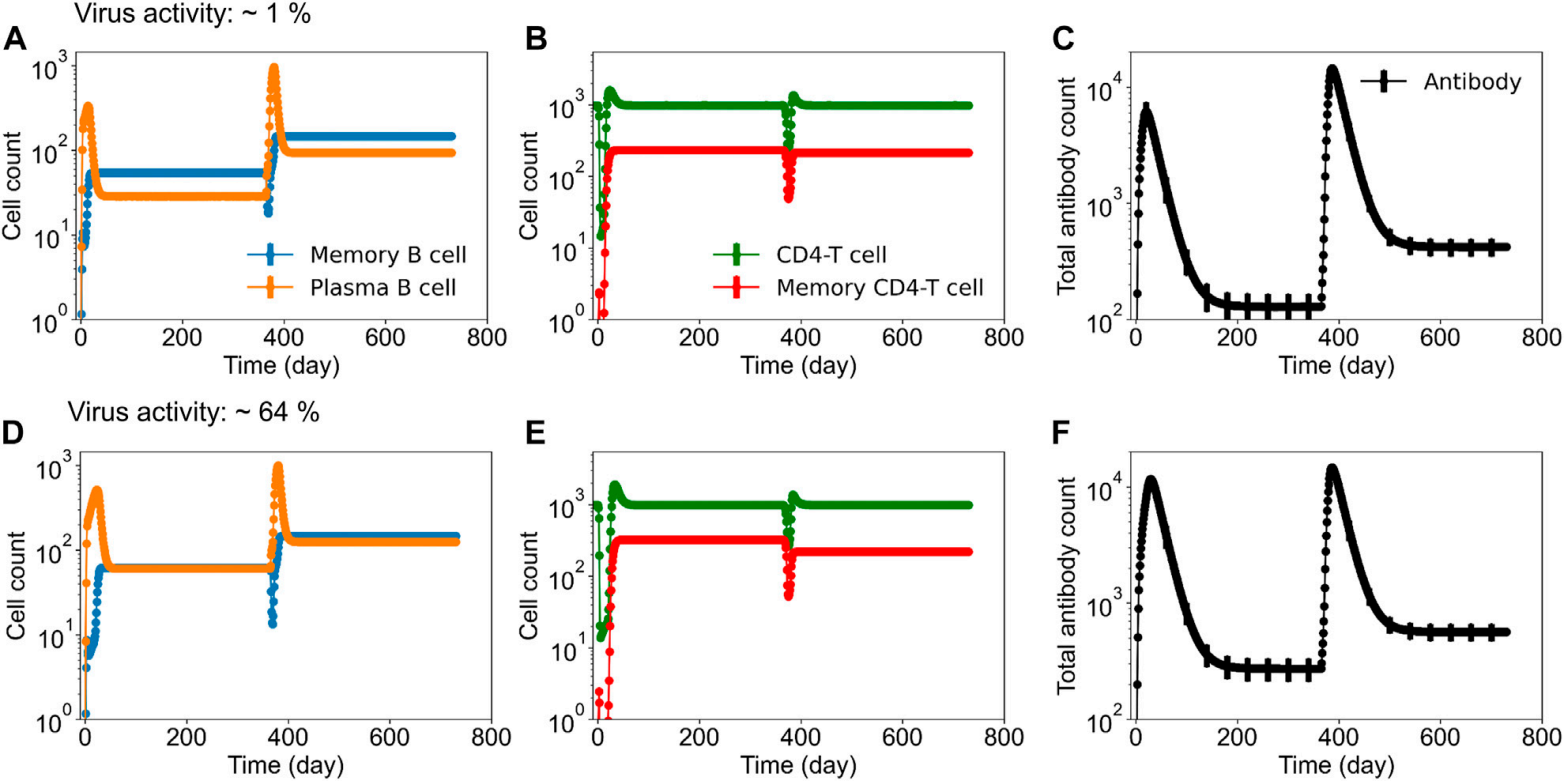
Stochastic B cell affinity maturation model simulations for the evolution of B cell, T cell, and total antibody responses post vaccination and post DENV challenge. Concentrations of (units × 10^3^/mL) **(A,D)** plasma and memory B cell counts, **(B,E)** total and memory CD4^+^ T cell counts, and **(C,F)** total antibody counts *versus* time are shown for low (∼1%) and high (64%) virus replication in the tetravalent vaccine construct.

To identify tetravalent vaccine-induced alterations in immune responses, we further tested the production of serotype-specific antibodies post DENV-2 challenge. As expected, we saw a large increase in their production specific to the DENV-2 serotype (Figure 2B). However, we did not see any significant differences between their peak levels for different live-attenuation rates. To further quantify how the percent of virus replication in the live-attenuated vaccines affects immune responses post vaccination and post challenge, we gathered the stochastic simulations of the mean alterations in B and T cell populations. Figure 3 shows alterations in total B cell, CD4^+^ T cell, and total antibody populations (both serotype-specific and cross-reactive) for two different virus activities (1% and 64%) in the live-attenuated vaccine constructs. Our results showed a slightly elevated circulation of plasma B cells (Figures 3A, D), memory CD4^+^ T cells (Figures 3B, E), and total antibody concentration (Figures 3C, F) in the pre-challenge phase for the live-attenuated constructs with the higher percent of virus replication (64%) as compared to the lower one (1%). These results emphasize the role of memory CD4^+^ T cells in the activation of the B cell affinity maturation process and the subsequent B cell differentiation into plasma B cells that produce the neutralizing antibodies for protection in the presence of DENV.

### DENV protection by tetravalent vaccination depends on the extent of virus attenuation

Based on the observations from previous studies that correlate peak viral titers from patients with secondary heterotypic infections to disease severity (Vaughn et al., 2000; Salje et al., 2018), we classified the secondary viral peaks into four different disease categories (Nguyen et al., 2021). Using k-means clustering, we divided the secondary viral peak populations into non-symptomatic (viral count below 5,600), mild (5,600–11,000), moderate (11,000–25,000), and severe cases (above 25,000). Using these disease classification criteria, we tested the tetravalent live-attenuated vaccine constructs for their efficacy in providing protection against a secondary DENV challenge (DENV-2) for the dengue-naive population. Table 1 shows the mean and standard deviation of secondary peak viremia values as well as the average percentage of the population affected by each disease severity (from a population size of 10,000) for different virus activities in the live-attenuated vaccine constructs. Our results clearly showed a reduced mean peak viremia and disease severity with increased virus replication in the vaccine construct. For example, our simulations showed that the population who experienced non-symptomatic cases would improve to 64% (from 44%), and severe cases would decrease to 0.16% (from 0.63%) if the virus replication was increased from 1% to 64% in the live-attenuated vaccine construct. Furthermore, the mild and moderate cases also significantly decreased with an increased virus replication in the vaccine constructs. These results suggest the importance of prior infection (or percent viral attenuation in the vaccine) and viral load required to develop protective immune responses.

**TABLE 1:** Average percentage of populations classified by disease severity, based on secondary peak viremia (DENV-2) for different virus activities in the live-attenuated vaccine construct.

Virus replication (%)	Peak viremia		Disease severity classification (%)			
	Mean	SD	Non-symptomatic	Mild	Moderate	Severe
0.80	7,024	4,695	44.73	43.02	11.61	0.63
5.70	6,344	3,932	51.76	39.60	8.20	0.44
13.30	6,051	3,549	55.51	37.00	7.30	0.23
21.42	5,848	2,784	58.06	35.50	6.11	0.31
37.00	5,610	3,406	62.22	31.63	5.86	0.27
64.00	5,469	3,237	63.60	31.12	5.10	0.16

Values were calculated using a population size of 10,000 and a tetravalent live-attenuated vaccine construct. SD, standard deviation.

The four serotypes of dengue virus share 65% sequence similarity. Although we are using DENV-2 serotype as the secondary DENV challenge model as it is the most prevalent serotype causing severe disease (Yung et al., 2015), we tested to see if differences in serotypes at the sequence level would alter the disease severity conditions. For example, we tested DENV-3 and DENV-4 as the subsequent challenge DENV serotypes after a single dose of vaccination. We did not see a major difference with respect to disease severity using DENV-3 as the challenge model compared to DENV-2 (results not shown). However, we did see noticeable differences when using DENV-4 as the challenge model. Our results show that the mean peak viremia decreased to 5,338 (SD: 2,674) compared to DENV-2 challenge model (mean: 6,051; SD: 3,549) for 13% viral attenuation rates. The corresponding percentage of population affected by each disease severity after DENV-4 challenge considerably improved, with non-symptomatic cases increasing to 63% and symptomatic cases decreasing for mild, moderate, and severe cases to 32.5%, 3.7%, and 0.042%, respectively (Supplementary Figure S3A). These results suggest that based on differences in DENV serotypes at the sequence level, the DENV-4 serotype was more efficiently neutralized with better antibody-mediated neutralization capabilities directed against it.

**FIGURE 4:**
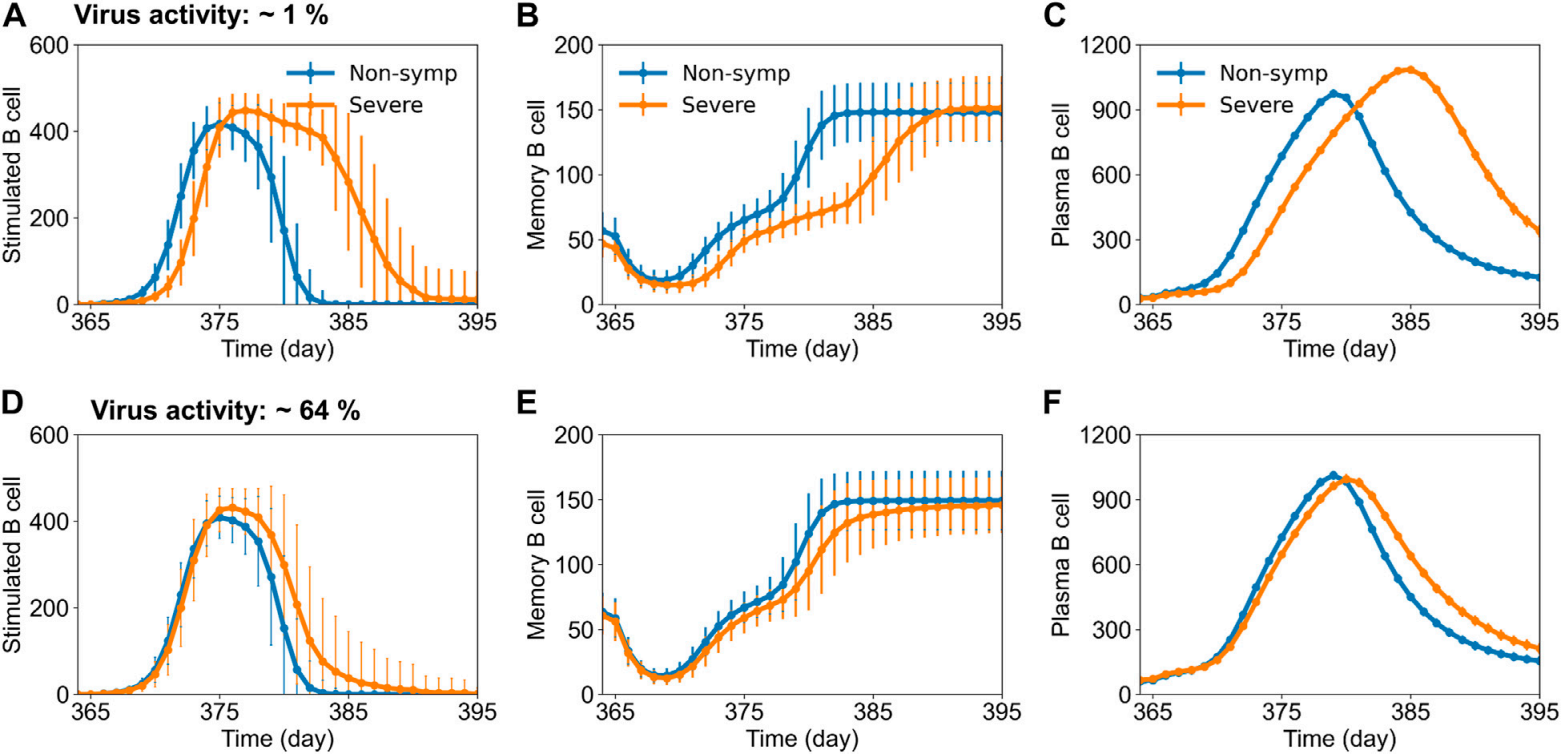
Stochastic B cell affinity maturation model simulations for the evolution of B cell responses post DENV-2 challenge for non-symptomatic and severe cases. Concentrations of (units × 10^3^/mL) **(A,D)** stimulated, **(B,E)** memory, and **(C,F)** plasma B cell counts *versus* time are shown for low (∼1%) and high (64%) virus replication in the tetravalent vaccine construct.

To further ascertain the mechanisms of protection imparted by vaccination, we compared the dengue-specific immune responses between disease classifications of non-symptomatic and severe cases for the DENV-2 challenge model. We compiled the cases from each disease severity population and calculated the mean alterations in the specific subtypes of B cell, T cell, and antibody counts across the population for each day. Figure 4 shows the mean alterations in different B cell populations post DENV-2 challenge for 1% (Figures 4A–C) and 64% (Figures 4D–F) virus replication in the live-attenuated vaccine construct. Our results showed that at the low virus replication, the stimulated, memory, and plasma B cell counts in circulation post DENV-2 were delayed in severe cases compared to the population with no symptoms, suggesting hampered immune responses leading to severe disease. However, these differences were significantly reduced when the virus replication was increased to 64%, indicating there are several other factors involved in the development of severe disease. For example, we also looked at the mean alteration in the T cell and antibody counts post DENV-2 challenge (Supplementary Figure S2) between non-symptomatic and severe cases. Although we observed a similar trend as that for B cells, the circulation of stimulated CD8^+^ T cell counts was consistently lower in severe disease cases for both the low and high virus replication live-attenuated vaccine constructs, indicating their prominent role in virus clearance.

**FIGURE 5:**
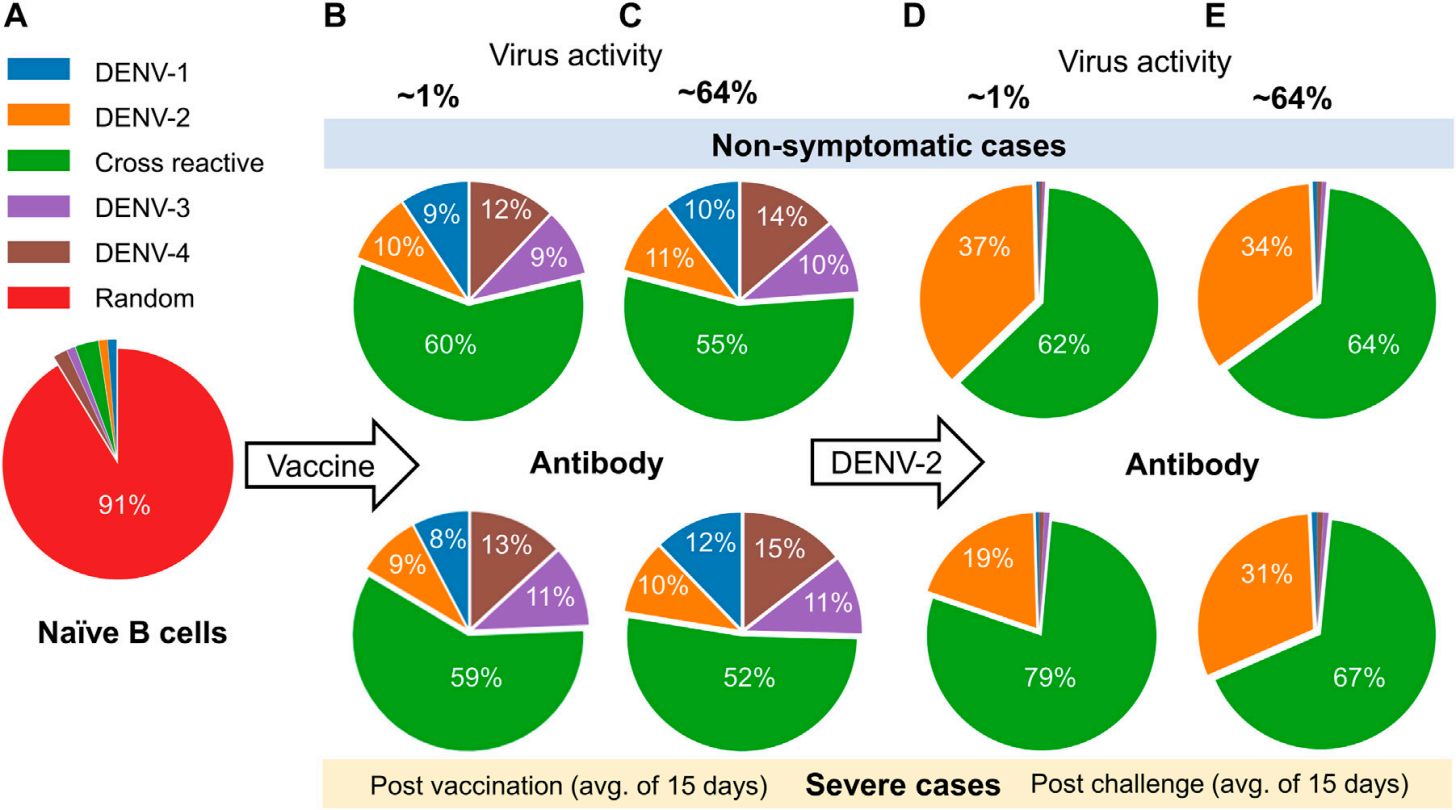
Analysis of the model simulations for the evolution of the percentage of type-specific antibody production between non-symptomatic and severe cases. **(A)** Percentage of naive B cells before vaccination. **(B–E)** Percentages of type-specific antibodies based on an average of 15 days **(B,C)** post vaccination and **(D,E)** post DENV-2 challenge for low (∼1%) and high (64%) virus replication in the tetravalent vaccine construct.

As it is known that the development of serotype-specific and cross-reactive antibodies correlates with the protection or lack of protection against DENV, we contrasted their profiles for non-symptomatic and severe cases between the low and high virus replication vaccine constructs (Figure 5). Most of the naive B cells before vaccination were random, with only a small percentage belonging to any particular serotype (Figure 5A). However, once a tetravalent vaccine was introduced into the system, we observed a roughly equal development of the four serotype-specific antibodies, but most of the remaining antibodies developed were cross-reactive (∼60%) and had roughly equal percentages for non-symptomatic and severe cases (Figures 5B, C). In contrast, after DENV-2 challenge, we observed a significant increase in the production of DENV-2-specific antibodies compared to other serotypes (Figures 5D, E). Specifically, production of serotype-specific antibodies was higher in non-symptomatic cases compared to severe cases, suggesting their protective role in non-symptomatic cases. Similarly, we observed that the increased production of cross-reactive antibodies for severe cases coincided with the reduced number of serotype-specific antibodies in both low and high virus replication vaccine constructs, although the difference was much smaller when the virus replication was high (Figure 5E). These results emphasize the protective role of serotype-specific antibodies and show that a minor imbalance in their production in comparison to cross-reactive antibodies may lead to severe dengue disease.

### A multiple-dose regimen provides better protection for the dengue-naïve population

The stochastic simulations performed using a single dose of tetravalent vaccine construct yielded simultaneous production of serotype-specific antibodies across all four serotypes. However, when we tested their efficacy after DENV-2 challenge, we saw that only 64% of the population was protected with no symptoms at high virus replication (Table 1). Therefore, we tested the effect of multiple doses of tetravalent live-attenuated vaccine constructs in providing protection against DENV. To this end, we introduced a tetravalent vaccine with 13% virus replication into the system on days 0, 90, and 180 then subjected the population to a DENV-2 challenge 1 year after the last dose. As expected, we observed a large improvement: 90% of the dengue-naïve population showed no symptoms, and symptomatic cases decreased drastically to 8%, 1%, and 0.016% for mild, moderate, and severe cases, respectively. These results suggest the effectiveness of a multiple-dose regimen in providing protection for the dengue-naïve population compared with a single-dose tetravalent vaccine. Furthermore, when we compared these disease severity outcomes for the three-dose regimen against no vaccination, we identified a 60% reduction in the mild symptomatic cases and 85% reduction in the moderate and severe symptoms. These results qualitatively agree with the Dengvaxia clinical trials, where they observed 40%–60% of the population being protected against dengue fever and reduced hospitalizations by 81% under similar conditions (Hadinegoro et al., 2015; Thomas and Yoon, 2019).

**FIGURE 6:**
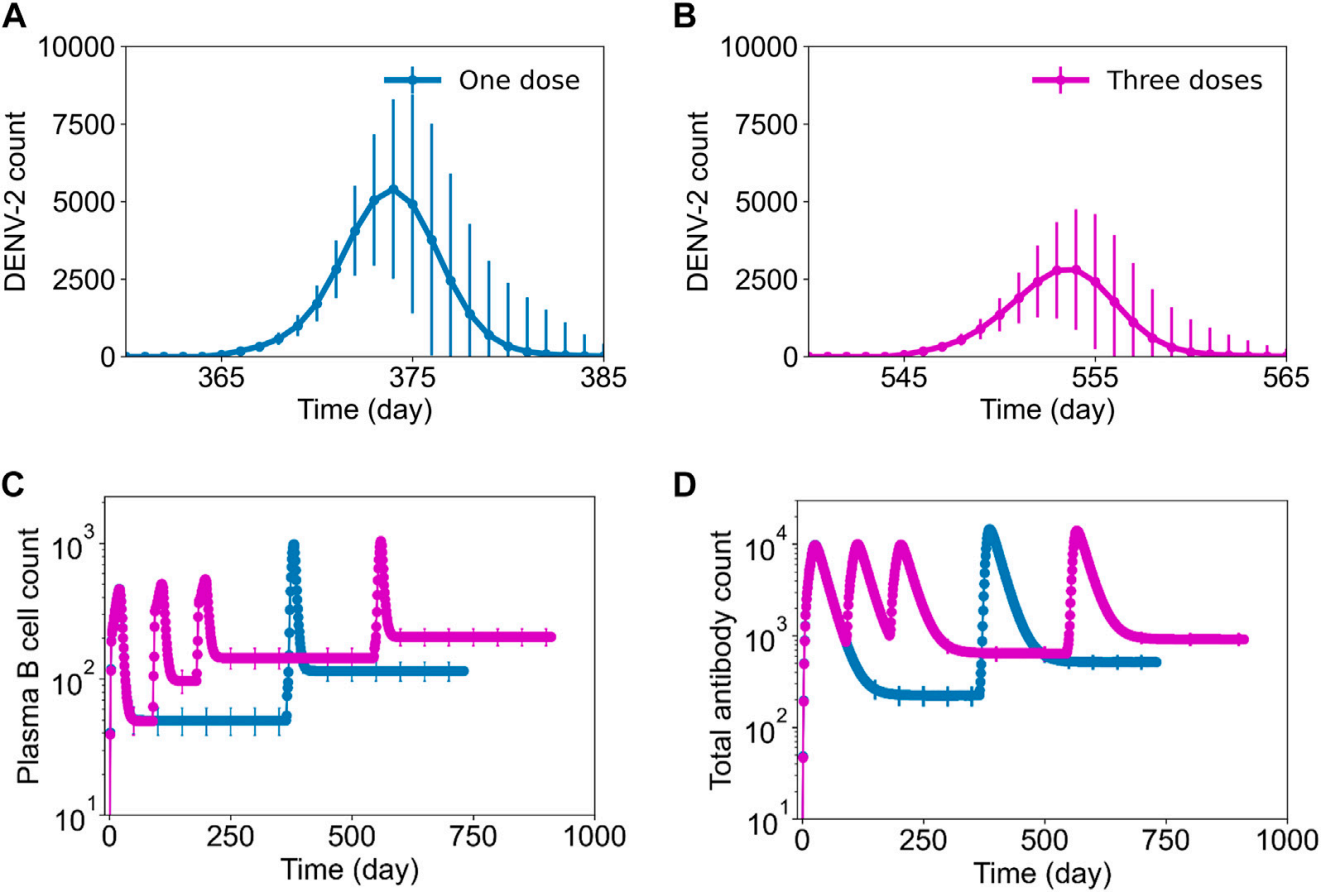
Stochastic B cell affinity maturation model simulations for DENV growth kinetics post challenge and B cell dynamics with single-dose and three-dose regimens. Average concentration (units × 10^3^/mL) of virus particles as a function of time post DENV-2 challenge is shown for **(A)** a single-dose regimen and **(B)** a three-dose regimen. Concentrations of (units × 10^3^/mL) **(C)** plasma B cell counts and **(D)** total antibody counts post a single dose of vaccination on day 0 (blue line) and post three-doses on 0, 90, and 180th day (pink line) followed by a DENV-2 challenge after one-year for each condition.

To understand the mechanisms behind a multiple-dose regimen providing protection, we captured the virus growth kinetics after DENV-2 challenge and the evolution of plasma and total antibody counts during the infection (Figure 6). Our results show that secondary virus growth was significantly hampered in the presence of multiple doses (Figure 6B) compared to a single-dose tetravalent vaccine (Figure 6A). Furthermore, the production of plasma B cells with a multiple-dose regimen yielded marginally higher peak values, but their basal levels before DENV-2 challenge were significantly higher compared to a single-dose vaccination (Figure 6C). We observed a similar behavior for total antibodies, with a multiple-dose regimen producing higher basal levels of protective antibodies before challenge and leading to better overall protection (Figure 6D).

## Discussion

Dengue circulates as four different serotypes, and they cross-react immunologically with each other. Furthermore, one of the peculiar features of dengue is that infection with one serotype provides long-lasting homotypic protection but enhances the severity of a subsequent heterotypic infection, leading to severe dengue disease (Halstead, 1989; Thomas, 2015; Gallichotte et al., 2018). To date, there is no specific treatment for dengue fever, and the main preventive measure is vaccination to reduce the burden of the disease. One way in which the dengue vaccines were developed was using live-attenuated viruses. Live-attenuated vaccines contain virus particles that are attenuated to be less virulent or even avirulent (Whitehead et al., 2007). Among the live-attenuated vaccines, Dengvaxia was developed to be a vaccine with four live chimeric viruses independently replicating and stimulating immunity to each DENV serotype. However, the DENV-4 component of the vaccine is more dominant than the other three components, therefore not inducing a balanced immunity (Chambers et al., 1999; Guy et al., 2009; Thomas and Yoon, 2019). Similarly, another tetravalent live-attenuated vaccine, developed by Takeda Vaccines, was based on the DENV-2 strain as its genetic backbone and induced higher levels of neutralizing antibodies to only DENV-2 compared to the other three serotypes in dengue-naïve populations (Biswal et al., 2019).

In comparison to the two live-attenuated vaccines described above, the TV-003 vaccine, developed by the United States National Institutes of Health, integrated the four monovalent vaccines with the live-attenuated tetravalent vaccine (Durbin et al., 2011). As a result, the vaccine has a balanced immune response against all four DENV serotypes (Simasathien et al., 2008; Nivarthi et al., 2021). These observations indicate that independent replication of each vaccine component above a threshold level is important for protection, and we need a better understanding of the mechanisms of these tetravalent live-attenuated vaccines in providing protective immune responses across four serotypes in dengue-naïve populations. In this work, we used our previously developed computational model of B cell affinity maturation to study the immune responses to different *in silico* tetravalent live-attenuated vaccine constructs and used the simulation results to understand the evolution of protective immune features for dengue-naïve populations.

Based on the model’s capability to simulate viral growth kinetics during natural infections, including the lag time for virus incubation, peak viremia range, and clearance rate, we computationally achieved an attenuated virus with altered virus growth kinetics representing the live-attenuated vaccine constructs. Furthermore, the computational framework gave us the opportunity to understand the development of serotype-specific and cross-reactive antibody responses post vaccination by keeping the virus replication rate the same for all four serotypes in the vaccine. As expected, the model simulations for the viral growth kinetics post vaccination indeed showed an equal viremia for all four serotypes, and the peak viremia was dependent on the viral attenuation rates (Figure 1). However, when we tracked the production of serotype-specific antibodies immediately post vaccination, we observed a slight advantage for DENV-4, with marginally higher peak antibody production compared to the other three serotypes (Supplementary Figure S1), indicating its unique characteristics based on differences at the epitope level between the serotypes. In addition, our observations for the peak serotype-specific antibody levels indicated a logarithmic correlation between peak antibody production and the level of viral replication in the vaccine, with peak values reaching a plateau above a certain threshold replication (Figure 2A). These results indicate the importance of vaccine viral replication for producing optimal levels of protective immune responses that play a crucial role in providing better protection from subsequent secondary dengue infections. For example, the post vaccination basal levels of memory CD4^+^ T cell, plasma B cell, and total antibody population were significantly higher in a live-attenuated vaccine construct with a higher *versus* lower virus replication in the vaccine (Figure 3).

Our analyses, based on disease classification into different severity levels as determined by secondary peak viremia, helped us to quantify the percentage of subjects who fell into the protected category with no symptoms to severe dengue disease with observable symptoms (mild, moderate, and severe). Assuming the population of subjects with mild to no symptoms as protected, we predicted that 93% of population would be protected from symptomatic disease, 1 year after a single dose of tetravalent live-attenuated vaccine with high virus replication post DENV-2 challenge (Table 1). Furthermore, a comparison of non-symptomatic and severe cases after DENV challenge indicated that the population with severe symptoms had delayed B cell (memory and plasma B cells) and T cell (memory CD4^+^ and stimulated CD8^+^) levels compared to non-symptomatic cases (Figure 4; Supplementary Figure S2). However, these differences were much smaller when we compared these B and T cell populations with the simulations for severe cases with natural infections (DENV-1 as primary infection followed by DENV-2) (Nguyen et al., 2021), suggesting the effectiveness of tetravalent live-attenuated vaccines in stimulating a balanced immune response.

As noted above, we observed a slightly higher peak serotype-specific antibody production post vaccination for DENV-4 for any tetravalent vaccine construct irrespective of the viral replication rate (Supplementary Figure S1) indicating serotype-dependent response to DENV. Therefore, we tested how disease severity would be affected in the case of a subsequent DENV-4 challenge instead of DENV-2. We observed a much-reduced disease severity for DENV-4 at a 13% viral replication in live-attenuated vaccine construct implying better protection for DENV-4 serotype compared to others. We indeed observed a higher production of DENV-4 specific antibodies post challenge when we used DENV-4 as the challenge model (Supplementary Figure S3) resulting in a more efficient neutralization of DENV-4 infections compared to other serotypes. Our results match with the clinical observations from a prospective study that DENV-2 serotype was the most prevalent with 57% of the cases with severe disease compared to 3.8% for DENV-4 (Yung et al., 2015).

For tetravalent dengue vaccines, the development of total neutralizing antibodies to each serotype alone may not be a reliable correlate of protection (Sridhar et al., 2018). It is believed that for dengue-naïve populations who received tetravalent live-attenuated vaccines, the serotype-specific antibodies independently stimulated by each vaccine component may be a better correlate of protection (Biswal et al., 2019; White et al., 2021). Our stochastic model simulations of type-specific and cross-reactive antibodies between the non-symptomatic and severe cases predicted a higher production of serotype-specific antibodies for non-symptomatic populations (Figure 5). These results indicate that serotype-specific antibodies could be a better correlate of protection, as was observed in some of the experimental studies using tetravalent live-attenuated vaccines (Kirkpatrick et al., 2016; Henein et al., 2021; Nivarthi et al., 2021). However, fact that the difference between the levels of these serotype-specific and cross-reactive antibodies between severe and non-symptomatic cases is very small at higher vaccine viral activities indicates that there is a delicate balance between them, together with the basal plasma B cell and T cell populations, in providing protection against DENV. Our simulations using multiple doses of tetravalent vaccine further confirmed these aspects of immune responses, with higher basal levels of plasma B cells and total antibodies (Figures 6C, D) leading to a weakened replication of secondary infection (Figure 6B) that resulted in a larger population being protected with no dengue symptoms as compared to a single dose. A further examination of serotype-specific antibody production after challenge showed that non-symptomatic populations who received three doses indeed had marginally lower DENV-2-specific antibodies compared to a single dose, indicating a role for higher basal levels of B cells, T cells, and total antibodies prior to infection in providing protection.

In conclusion, we used a stochastic model of B cell affinity maturation to study the humoral response to potential tetravalent live-attenuated constructs. Using model simulations of viral growth kinetics, we elucidated the level of virus replication needed in a vaccine to develop optimal levels of protective serotype-specific antibodies after vaccination. Our simulations further clarified the mechanism of protection for populations with no symptoms, with higher production of serotype-specific antibodies and lower cross-reactive antibodies as correlates of protection. In addition, our model simulations also captured additional mechanisms, such as higher basal levels of B and T cells pre- and post-secondary infection, as prerequisites for the observed protection against DENV. In addition, our model simulations also showed that the efficacy of tetravalent vaccine constructs fare better against the DENV-4 serotype compared to other serotypes due to its differences at the sequence level. Although our study provided valuable insights into the mechanism of protection mediated by tetravalent vaccines, our simulations were based on the initial B and T cell populations of 6- to 12-year-old children. Future studies with different age groups and using a more detailed representation of live-attenuated vaccine kinetics would help us to further understand the underlying mechanisms for development of protective immune responses against severe dengue and in the design of effective tetravalent dengue vaccines.

## Data Availability

The original contributions presented in the study are included in the article/Supplementary Material, further inquiries can be directed to the corresponding authors.
