# Supplementary material for: A stochastic B cell affinity maturation model to characterize mechanisms of protection for tetravalent dengue vaccine constructs
Source: Front Mol Biosci. 2023 Jul 14;10:1100434. doi: 10.3389/fmolb.2023.1100434 (PMC10375700; doi:10.3389/fmolb.2023.1100434)
Supplement: Supplementary file 1 [file Table1.DOCX]

**Supplementary Materials**

**A Stochastic B Cell Affinity Maturation Model to Characterize Mechanisms of Protection for Tetravalent Dengue Vaccine Constructs**

Venkat R. Pannala^1,2*^, Hung D. Nguyen^1,2^, and Anders Wallqvist^1*^

^1^Department of Defense Biotechnology High Performance Computing Software Applications Institute, Telemedicine and Advanced Technology Research Center, U.S. Army Medical Research and Development Command, Fort Detrick, MD, United States

^2^The Henry M. Jackson Foundation for the Advancement of Military Medicine, Inc., Bethesda, MD, United States

^*^Correspondence

Anders Wallqvist

Phone: 301-619-1989, Fax: 301-619-1983

E-mail: sven.a.wallqvist.civ@health.mil

Venkat R. Pannala

Phone: 301-619-1978, Fax: 301-619-1983

E-mail: vpannala@bhsai.org

**Supplementary Methods**

We modeled the B-cell affinity maturation of stimulation and proliferation with the aid of helper CD4^+^ T cells using a set of rate equations. The system contains stochastic equations for memory and plasma B cell differentiation, antibody (Ab) production, and virus clearance by either Abs or cytotoxic CD8^+^ T cells. In the system, we explicitly denoted the genotype of every virus epitope as subscript *i* that belongs to one of four virus serotypes (Table S1) and paratope of B cell receptor and antibody as subscript *j* in the rate equations.

We modeled the dengue virus replication using a first-order rate equation forming two copies from every dengue virus with rate constant *k_v_* and its subsequent intrinsic decay with rate constant *g_v_*. This non-specific clearance process is related to its half-life of dengue virus which is 5.2 hours at 37 ºC (1). The dengue virus replication and decay are modeled using following rate equations, with respect to any of the four dengue virus serotypes (*i)*:

$V_{i} \overset{\to}{k_{v}}2V_{i}$ (1a)

$V_{i}\overset{\to}{g_{v}} \phi$ (1b)

In this model, to maintain an equilibrium concentration of naïve B (N_j_; *j* is B-cell paratope) and helper T cells, we modeled them as continuously replenished variables and whose formation and decay are described by zero-order and first-order rate equations, respectively. The initial concentrations of these variables are randomly chosen for every simulation from a non-normal distribution whose median, minimum, and maximum values are selected from a healthy population of 6-12 years old children (2).

$\phi\overset{\to}{k_{N}} N_{j}$ (2a)

$N_{j} \overset{\to}{g_{N}}\phi$ (2b)

$\phi\overset{\to}{k_{T1}} T_{CD4}$ (2c)

$T_{CD4} \overset{\to}{K_{T2}}\phi$ (2d)

$\phi\overset{\to}{k_{T3}} T_{CD8}$ (2e)

$T_{CD8} \overset{\to}{K_{T4}}\phi$ (2f)

We modeled the dengue virus mediated naïve B-cell migration into germinal center B-cells and stimulated B cells (B_j_*) as second-order rate equations.

$N_{j} + V_{i}\overset{\to}{\sigma_{N}\gamma_{i}Q_{ij}}B_{j}+V_{i}$ (3a)

$B_{j} + V_{i}\overset{\to}{\sigma_{N}\gamma_{i}Q_{ij}}B_{j}^{*}+V_{i}$ (3b)

Subsequently, we modeled the stimulated B cell (B*) conversion into activated B cells (B**) with the help of helper CD4^+^ T cells using a second-order rate equation with rate constant *k_T5_*_­_.

$B_{j}^{*} + T_{CD4}\overset{\to}{k_{T5}}B_{j}^{**}+T_{CD4}^{*}$ (4)

Once activated by helper T cell, the activated B cells undergo proliferation and differentiation into memory (M_j_) and plasma B cells (short lived: P^s^_j_ and long-lived: P^L^_j_) as described below. Here, $\alpha$ denotes the probability of activated B cell conversion into either short or long-lived plasma cells.

$B_{j}^{**} \overset{\to}{{rR}_{jk}}B_{j}+B_{k}$ (5a)

$B_{j}^{**} \overset{\to}{\delta}M_{j}$ (5b)

$B_{j}^{**} \overset{\to}{\delta}\alpha P_{j}^{S}+\left( 1-\alpha\right)P_{j}^{L}$ (5c)

$B_{j}^{**} \overset{\to}{max(\eta,g_{B})}\phi$ (5d)

Serum antibody (Ab) production from both short and long-lived plasma cells are modeled as below. Here, we assumed only short-lived plasma cells undergo a decay process.

$P_{j}^{S} \overset{\to}{k_{Ab}}P_{j}^{S}+{Ab}_{j}$ (6a)

$P_{j}^{L} \overset{\to}{k_{Ab}}P_{j}^{L}+{Ab}_{j}$ (6b)

$P_{j}^{S} \overset{\to}{k_{Ab}}\phi$ (6c)

In the presence of dengue virus, the memory cells that already present previously transform into plasma cells to provide an immediate Ab response without going through affinity maturation process starting from B cells. We modeled this scenario using a second-order rate equation as described below.

$M_{j}+ V_{i}\overset{\to}{\sigma_{M}\gamma_{i}Q_{ij}}P_{j}^{S}+P_{j}^{L}+V_{i}$ (7)

Similar to the activated B cells, activated CD4^+^ T cells undergo differentiation into either original CD4^+^ T cells or memory CD4^+^ T cells and decay to null subsequently. We modeled these transformations using a first-order rate equation with rate parameters *k_T6 to T8_*.

$T_{CD4}^{*} \overset{\to}{k_{T6}}T_{CD4}$ (8a)

$T_{CD4}^{*} \overset{\to}{k_{T7}}T_{CD4}^{M}$ (8b)

$T_{CD4}^{*} \overset{\to}{k_{T8}}\phi$ (8c)

We also included a rate equation for memory CD4^+^ T cells transformation back to CD4^+^ T cells and activated B cells using a second order rate equation and are not tagged for decay in the simulations.

$B_{j}^{*} + T_{CD4}^{M}\overset{\to}{k_{T9}}B_{j}^{**}+T_{CD4}^{*}$ (9)

Similarly, we modeled cytotoxic CD8^+^ T cells activation by activated CD4^+^ T cells through a second-order rate equation with rate constant *k_T10_* and activated CD8^+^ T cell transformation back to CD8^+^ T cells and their decay as described below.

$T_{CD4}^{*} + T_{CD8}\overset{\to}{k_{T10}}T_{CD4}^{*}+T_{CD8}^{*}$ (10a)

$T_{CD8}^{*} \overset{\to}{k_{T12}}T_{CD8}$ (10b)

$T_{CD8}^{*} \overset{\to}{k_{T13}}\phi$ (10c)

Finally, we modeled the dengue virus clearance by antibodies and activated cytotoxic CD8^+^ T cells using second-order rate equations and the antibody decay using a first-order rate equation as described below.

${Ab}_{j} + V_{i}\overset{\to}{\rho_{i}Q_{ij}}{Ab}_{j}$ (11a)

$T_{CD8}^{*}+ V_{i} \overset{\to}{k_{T11}}T_{CD8}^{*}$ (11b)

${Ab}_{j} \overset{\to}{g_{Ab}}\phi$ (11c)

We provide all the rate parameters relevant B-cell affinity maturation process in the Supplementary Table S2 and various altered dengue virus replications rates for tetravalent vaccine constructs in the Table S3 below.

Table S1: Sequences and parameter values for each epitope of each serotype

| Serotype | Epitope | Sequence |
| --- | --- | --- |
| 1 | PrM | aaaa aaaa aaaa aaaa aaaa |
| 1 | FL | bbbb bbbb bbbb bbbb bbbb |
| 1 | DIII | cccc cccc cccc cccc cccc |
| 1 | hinge | dddd dddd dddd dddd dddd |
| 2 | PrM | aaaa aaaa aaaa aaaa aaaa |
| 2 | FL | bbbb bbbb bbbb bbbb bbbc |
| 2 | DIII | cccc cccc cccc cccc dddd |
| 2 | Hinge | dddd dddd dddd ddda aaaa |
| 3 | PrM | aaaa aaaa aaaa aaaa aaaa |
| 3 | FL | bbbb bbbb bbbb bbbb bbbd |
| 3 | DIII | cccc cccc cccc cccc aaaa |
| 3 | hinge | dddd dddd dddd dddb bbbb |
| 4 | PrM | aaaa aaaa aaaa aaaa aaaa |
| 4 | FL | bbbb bbbb bbbb bbbb bbba |
| 4 | DIII | cccc cccc cccc cccc bbbb |
| 4 | hinge | dddd dddd dddd dddc cccc |

Table S2: Parameter values for the immune system model

| Model parameter | Symbol | Value | |
| --- | --- | --- | --- |
| **Simulation conditions** | | | |
| Virus dose | | | 10^5^ copies/ml |
| **Virus parameters** | | | |
| Serotypes | | 4 | |
| Epitopes | | 4 | |
| Epitope PrM | | | |
| Immunogenicity | γ | 0.85 | |
| Clearance | ρ | 0.1 | |
| Antigenic distance | | 0 | |
| Epitope FL | | | |
| Immunogenicity | γ | 1.50 | |
| Clearance | ρ | 1.0 | |
| Antigenic distance | | 1 | |
| Epitope DIII | | | |
| Immunogenicity | γ | 1.15 | |
| Clearance | ρ | 1.0 | |
| Antigenic distance | | 4 | |
| Epitope hinge | | | |
| Immunogenicity | *γ* | 1.50 | |
| Clearance | *ρ* | 1.0 | |
| Antigenic distance | | 5 | |
| Virus formation rate | *k_V_* | 6.2 ml/(copies x d) | |
| Virus decay rate | *g_V_* | (8.0 h)^-1^ | |
| **B cell parameters** | | | |
| B cell enhancement factor | *ε_B_* | 10 | |
| Ab enhancement factor | *ε_Ab_* | 2.5 | |
| Naïve B cell formation rate* | *k_N_* | (R h)^-1^ | |
| Naïve B cell stimulation | *σ_N_* | (1 d)^-1^ | |
| GC B cell stimulation (base) | *σ_base_* | (8 h)^-1^ | |
| GC B cell stimulation (maximum) | *σ_max_* | (15 min)^-1^ | |
| GC B cell replication rate | *r* | (8 h)^-1^ | |
| Mutation probability | *μ* | 0.10 | |
| Differentiation probability | *δ* | 0.10 | |
| Memory cell stimulation | *σ_M_* | (1 d)^-1^ | |
| Ab production | *k_Ab_* | 1.0 | |
| Naïve B cell decay rate | *g_N_* | (4.5 d)^-1^ | |
| GC B cell decay rate (base) | *g_B_* | (4.5 d)^-1^ | |
| Plasma cell decay rate | *g_P_* | (3 d)^-1^ | |
| Ab decay rate | *g_Ab_* | (10 d)^-1^ | |
| **T cell parameters** |  |  | |
| CD4+ T cell formation rate* | *k_T1_* | R copies x (ml x d)^-1^ | |
| CD4+ T cell decay rate | *k_T2_* | (4.5 d)^-1^ | |
| CD8+ T cell formation rate* | *k_T3_* | R copies x (ml x d)^-1^ | |
| CD8+ T cell decay rate | *k_T4_* | (4.5 d)^-1^ | |
| CD4+ T cell activation rate | *k_T5_* | 1200 ml x (copies x h)^-1^ | |
| CD4+ T cell differentiation rate | *k_T6_* | (15 h)^-1^ | |
| Memory CD4^+^ T cell formation rate | *k_T7_* | (15 h)^-1^ | |
| Activated CD4^+^ T cell decay rate | *k_T8_* | (4.5 d)^-1^ | |
| Memory CD4^+^ T cell reactivation rate | *k_T9_* | 2400 ml x (copies x h)^-1^ | |
| CD8^+^ T cell activation rate | *k_T10_* | 8 ml x (copies x h)^-1^ | |
| CD8^+^-based clearance rate | *k_T11_* | 0.000025 ml x (copies x d)^-1^ | |
| CD8^+^ T cell differentiation rate | *k_T12_* | (180 h)^-1^ | |
| Activated CD8^+^ T cell decay rate | *k_T13_* | (4.5 d)^-1^ | |

*R: the rate constant corresponds to either naïve B cell, CD4^+^, or CD8^+^ concentration that was randomly chosen for every simulation from a non-normal distribution of a healthy population of 6-12 years old children.

Table S3: Parameters altered to simulate live-attenuated vaccine constructs

| Model parameter | Symbol | Value |
| --- | --- | --- |
| CD4+ T cell activation rate | k_T5_ | 400 ml x (copies x h)^-1^ |
| Memory CD4^+^ T cell reactivation rate | k_T9_ | 160 ml x (copies x h)^-1^ |
| CD8^+^ T cell activation rate | k_T10_ | 640 ml x (copies x h)^-1^ |
| CD8^+^-based clearance rate | k_T11_ | 0.000075 ml x (copies x d)^-1^ |
| Virus replication rate | k_V_ for 1% activity | 6.90 ml/(copies x d) |
| Virus replication rate | k_V_ for 6% activity | 6.25 ml/(copies x d) |
| Virus replication rate | k_V_ for 13% activity | 6.00 ml/(copies x d) |
| Virus replication rate | k_V_ for 21% activity | 5.90 ml/(copies x d) |
| Virus replication rate | k_V_ for 37% activity | 5.80 ml/(copies x d) |
| Virus replication rate | k_V_ for 64% activity | 5.70 ml/(copies x d) |

**
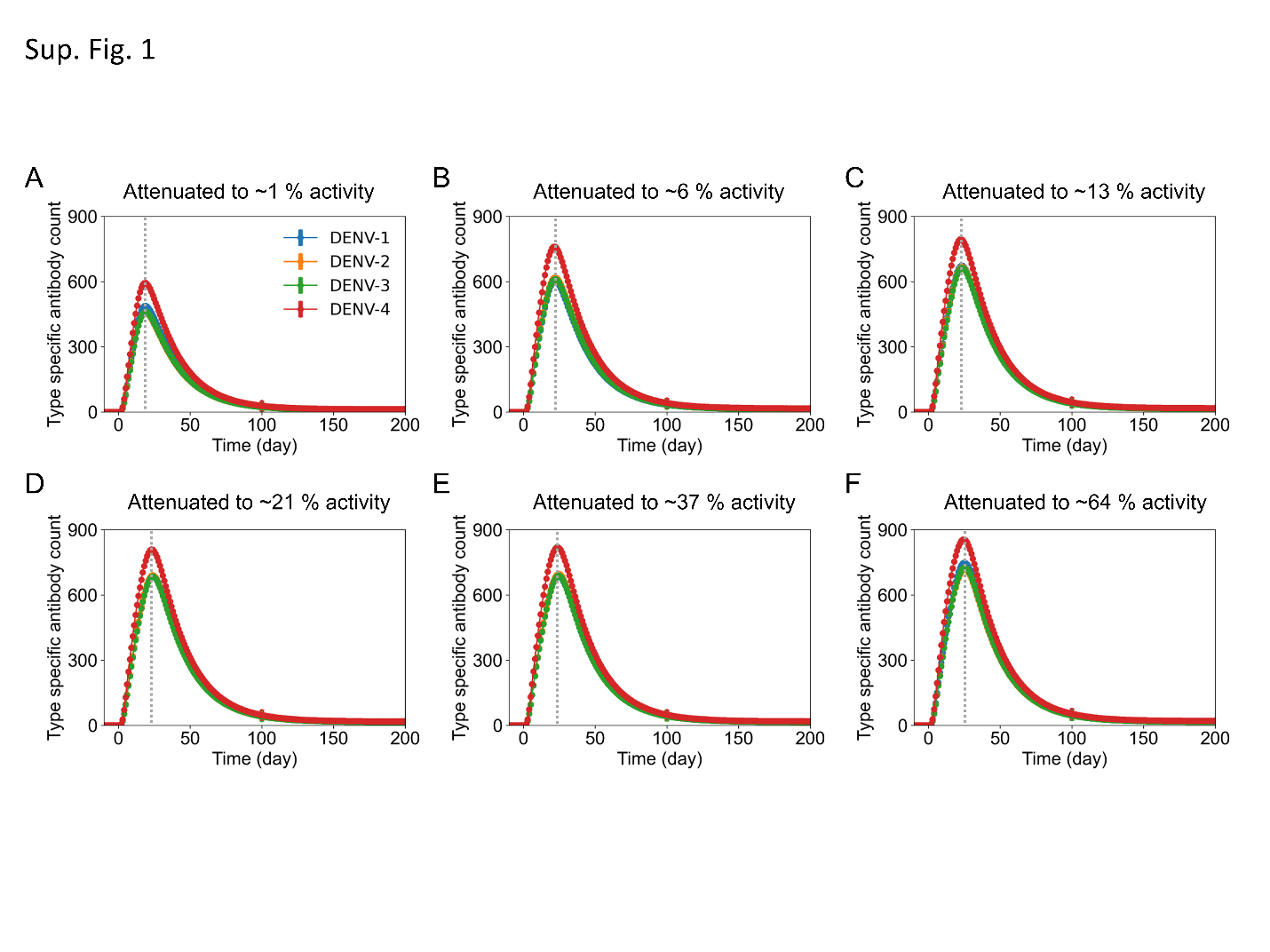
**

**Supplementary Figure 1:** Stochastic model simulations that produce serotype-specific antibodies post a single dose of vaccination (day zero) with the live-attenuated virus with different percentages of virus activity in the vaccine. Average concentration (units × 10^3^/ml) of serotype-specific antibody counts as a function of time post vaccination is shown for all four DENV serotypes at different virus replication rates, with virus activity percentages of (A) ~1%, (B) ~6%, (C) 13%, (D) ~21%, (E) ~37%, and (F) 64% as compared to virus replication with natural infections.

**
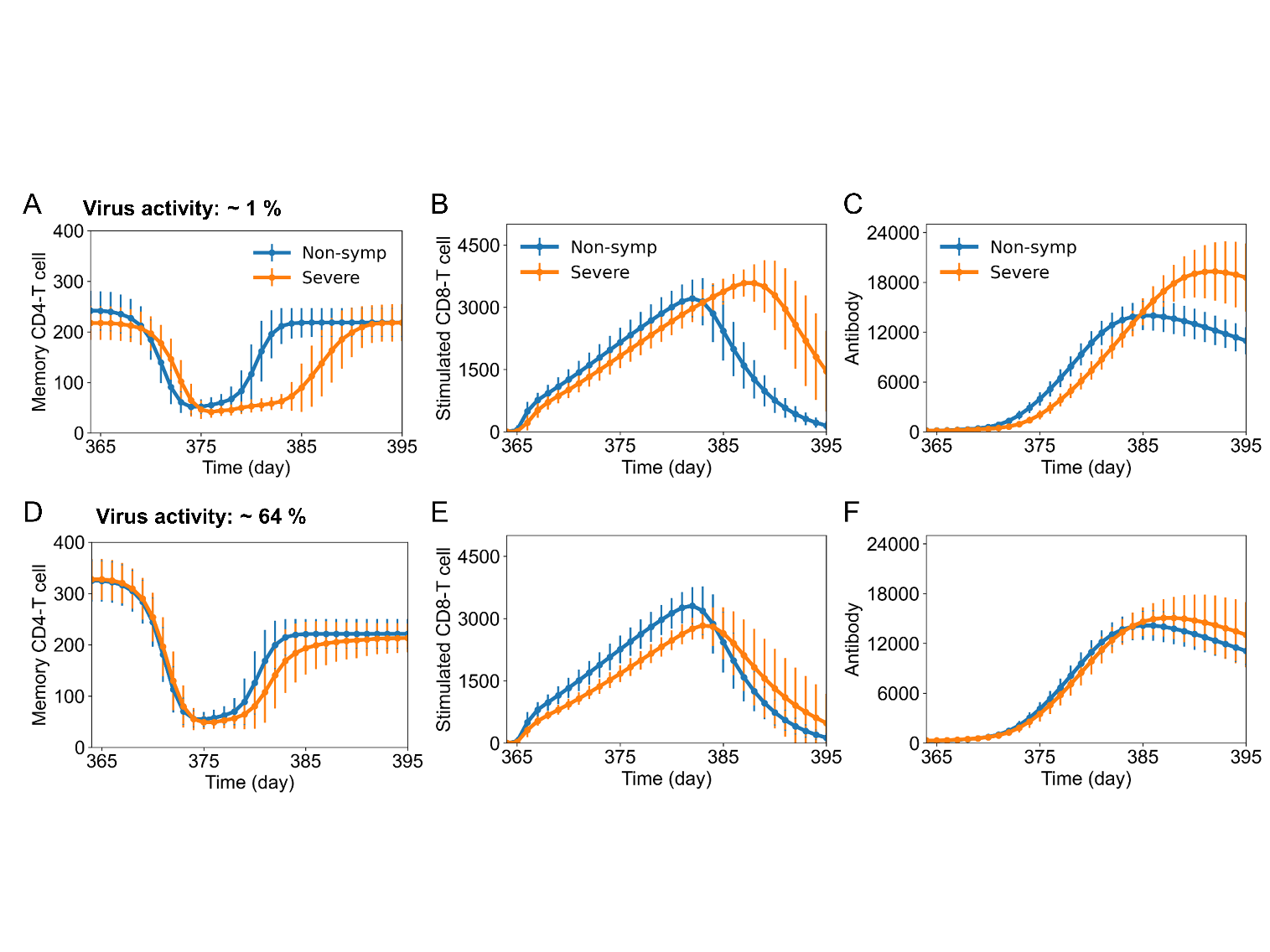
**

**Supplementary Figure 2:** Stochastic B cell affinity maturation model simulations for the evolution of T cell responses post DENV-2 challenge for non-symptomatic and severe cases. Concentrations of (units × 10^3^/ml) (A, D) memory CD4^+^ T cell counts, (B, E) stimulated CD8+ T cell counts, and (C, F) antibody counts versus time are shown for low (~1%) and high (64%) virus activity in the tetravalent vaccine construct.

**
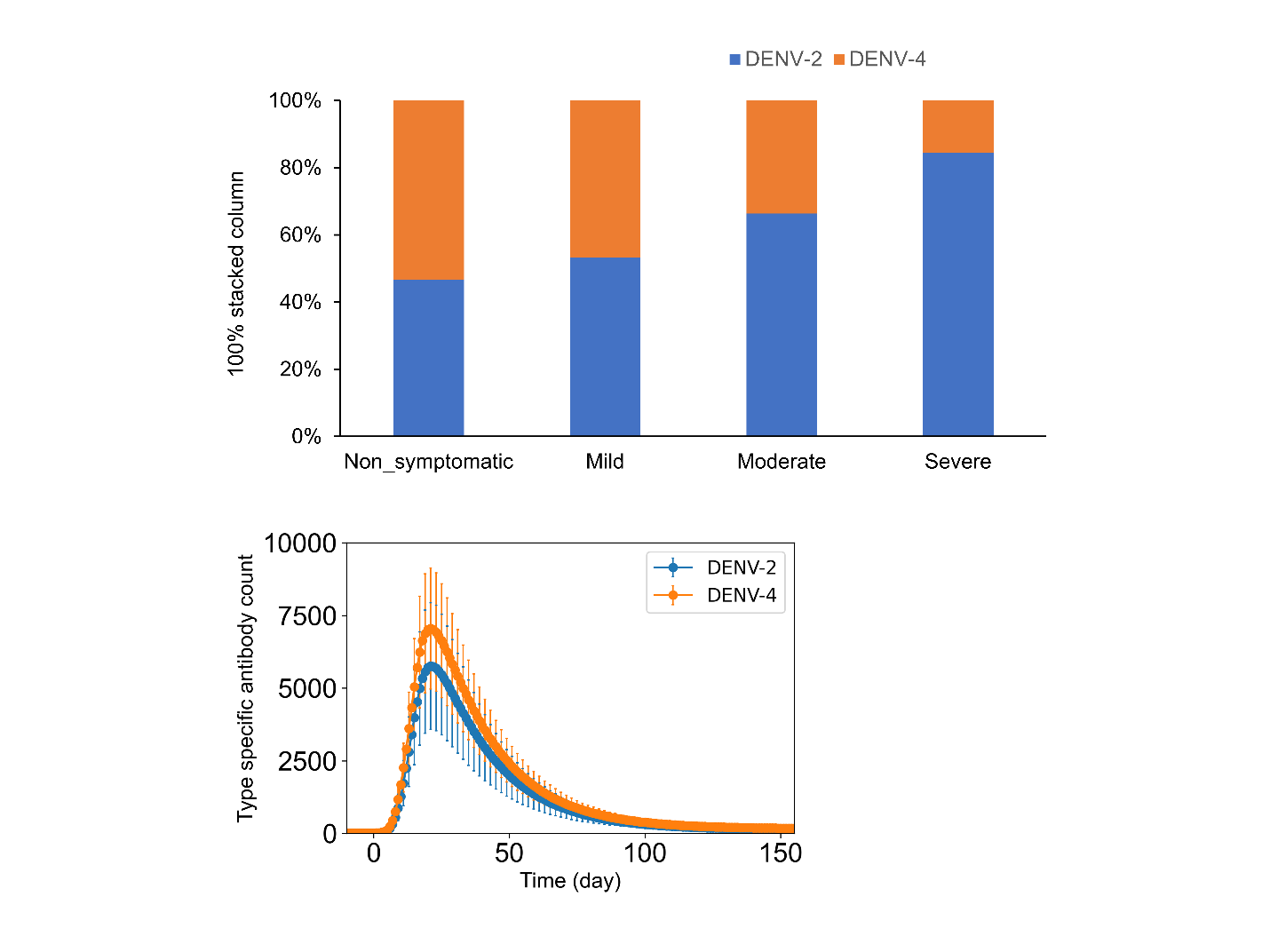
A)**

**B)**

**Supplementary Figure 3:** Stochastic B cell affinity maturation model simulations for the disease severity and serotype-specific antibody production post DENV-2 or DENV-4 challenge 1 year after a single dose of vaccination with a tetravalent vaccine construct of 13% virus activity.

A) Disease classification based on secondary peak viremia. Percent contribution of disease severity cases for DENV-2 (blue) and DENV-4 (orange) challenge model. B) Average concentration (units × 10^3^/ml) of serotype-specific antibody counts as a function of time post DENV challenge 1 year after a single dose of vaccination.

References

1. J. S. Manning and J. K. Collins: Effects of cell culture and laboratory conditions on type 2 dengue virus infectivity. *J Clin Microbiol*, 10(2), 235-9 (1979) doi:10.1128/jcm.10.2.235-239.1979

2. F. Tosato, G. Bucciol, G. Pantano, M. C. Putti, M. C. Sanzari, G. Basso and M. Plebani: Lymphocytes subsets reference values in childhood. *Cytometry A*, 87(1), 81-5 (2015) doi:10.1002/cyto.a.22520
